# Dietary Inflammatory Index score and its association with body mass index, body fat percentage, body fat mass, and lipid profile in patients with multiple sclerosis

**DOI:** 10.55730/1300-0144.5681

**Published:** 2023-06-21

**Authors:** Seda KAYA, Zeynep UZDİL, Nitin SHIVAPPA, James R. HEBERT, Pınar SÖKÜLMEZ KAYA, Murat TERZİ

**Affiliations:** 1 Department of Nutrition and Dietetics, Faculty of Health Sciences, Ankara University, Ankara, Turkiye; 2 Department of Nutrition and Dietetics, Faculty of Health Sciences, Ondokuz Mayıs University, Samsun, Turkiye; 3 Department of Epidemiology and Biostatistics and the Cancer Prevention and Control Program, University of South Carolina, Columbia, South Carolina, USA; 4 Connecting Health Innovations, LLC, Columbia, South Carolina, USA; 5 Department of Neurology, Faculty of Medicine, Ondokuz Mayıs University, Samsun, Turkiye

**Keywords:** Multiple sclerosis, Dietary Inflammatory Index, nutrition, obesity, lipid profile

## Abstract

**Background/aim:**

Multiple sclerosis (MS) may cause modifications in body composition, particularly for body fat associated with obesity and some biochemical parameters such as lipid profiles. We investigated whether there is a link between the inflammatory contents of diets and body composition and lipid profiles in patients with MS.

**Materials and methods:**

This was a cross-sectional study that included 85 MS patients. The study data of the patients were collected in the Neurology Clinic of Ondokuz Mayıs University’s Health Practice and Research Center. The data included demographic characteristics; anthropometric measurements such as body weight, height, body mass index, waist circumference, hip circumference, body fat mass, body fat-free mass, and waist-hip ratio; and biochemical parameters such as high-density lipoprotein cholesterol (HDL-c), low-density lipoprotein cholesterol, triglyceride, and total cholesterol.

**Results:**

The body fat percentages of the patients were higher among those with proinflammatory diets (p < 0.05). Body fat percentage had a positive and very weak correlation with the Dietary Inflammatory Index (DII) score (rho = 0.206 and rho = 0.217, respectively; p < 0.05). HDL-c levels were higher in the group with high DII scores and there was a positive and weak correlation between HDL-c and DII scores (rho = 0.307, p < 0.05). Crude and adjusted linear regression models showed that the effect of HDL-c on DII scores was significant (p < 0.05).

**Conclusion:**

We showed that DII scores, associated with the inflammatory potential of the diet and proinflammatory diets, may be associated with adiposity in MS patients and can be used from a clinical point of view for assessment.

## 1. Introduction

Multiple sclerosis (MS) is a chronic, inflammatory, immune-mediated condition that damages nerve fibers and the myelin sheath and affects the brain and spinal cord [1,2]. It is often observed among women and it causes neurological disability in young adults [3]. Comorbid diseases may also emerge depending on causes such as disability, reduced movement and quality of life, and increased obesity, which develop along with the clinical course of the disease and have significant impacts on the financial, social, and psychological well-being of patients [4,5]. Obesity affects millions of adults and there is an increasing trend towards obesity in patients with MS. This situation is responsible for increasing economic and health burdens worldwide [6–8].

Multiple sclerosis, a T cell-mediated autoimmune disease, is characterized by an increase in proinflammatory factors [9]. Although the etiology of chronic inflammatory demyelination, which usually occurs during young adulthood, is not clear, it is believed to be the result of a complex interaction between genetic and environmental exposures, including diet [10,11]. It is known that chronic inflammation, characterized by inflammatory cytokines in the circulation and tissues, plays an important role in the development of various diseases in the central nervous system (CNS) [12]. In addition, chronic inflammation is an important factor for MS disease, which is characterized by demyelination in the CNS [13].

Recent research shows that dietary components are associated with inflammation and many diseases including cardiovascular disease, metabolic syndrome, and some types of cancer [14]. Therefore, responding to modifiable risk factors may play an important role in preventing inflammation. Low-grade systemic inflammation is characterized by the constant presence of proinflammatory cytokines with increased blood flow during injury, which is known to play an important role in the development of diseases and associated mortality [15]. It has been reported that healthy nutrition and protective dietary patterns, which are important factors among environmental factors, reduce the levels of low-grade inflammation and oxidative stress and also improve endothelial function [16,17]. Therefore, they may have potential importance as etiological factors or therapeutic tools in the treatment of inflammatory-based disorders [18].

Clinically, functional disorders associated with loss of muscle mass and increased fat mass are observed in MS patients [19]. In this sense, the accumulation of abdominal fat is particularly important since it directly contributes to the chronic inflammatory state of the disease mediated by the production of proinflammatory cytokines [20]. Dietary guidelines for MS patients may have the potential to alleviate the symptoms associated with MS, as interchangeable lifestyle factors such as dietary quality can affect the course of the disease [21–23]. Progress in understanding the mechanisms of the impact of diet on inflammation and the quantification of effects has been challenging, however. Recent studies have evaluated the impact of dietary habits on human health by following the classical approach of evaluating single foods (e.g., fish or dark green vegetables) and nutrients (e.g., flavonoids) or using a holistic dietary approach such as the Mediterranean diet [24,25]. In 2009, Cavicchia et al. proposed the Dietary Inflammatory Index (DII), based on research conducted beginning in 2004, as a way of assessing the inflammatory potential of diets [26]. In 2013, Shivappa et al. proposed a new refined literature-based DII [27]. Using the new formulation, higher DII scores were shown to be associated with disorders and diseases such as obesity, glucose intolerance, metabolic syndrome, and dyslipidemia [28–30]. A recent study revealed that there may be a relationship between the DII and MS [31]. To our knowledge, however, there are no studies in the literature evaluating the relationships between the DII and obesity and lipid profiles in patients with MS. Therefore, considering the importance of dietary habits as a set of modifiable MS risk factors that can directly and cost-effectively improve the clinical situation of patients, the aim of this study was to evaluate the associations between DII scores and body mass index (BMI), body fat percentage, body fat mass (BFM), and lipid profiles in patients with MS.

## 2. Materials and methods

### 2.1. Study design and subjects

This cross-sectional study evaluating 85 relapsing-remitting MS (RRMS) patients between the ages of 18 and 59 years was conducted between May and June 2020 in the Ondokuz Mayıs University Health Practice and Research Center’s Neurology Clinic (Neurology Department) in the city of Samsun. This center is an institution that most patients with MS in the north of Türkiye visit for diagnosis and treatment. Patients aged 18 and over who were diagnosed with MS according to McDonald’s criteria [32] and who presented to the outpatient clinic were contacted. The data of 85 patients who completed the study were analyzed at the end of the research process. The study population comprised patients who were assisted in the aforementioned health unit between May and June 2020, totaling 203 individuals. Power analysis was conducted using R 3.6.1 statistical software (www.r-project.org). The sample size was calculated to be at least 85 for alpha = 0.05, power = 0.83, and effect size = 0.3. In June, patients were contacted and data were collected. Patients were excluded if they had a non-RRMS clinical course of MS, experienced episodes, had chronic diseases such as diabetes or cardiovascular disease, had an active or previous history of malignancy, used nutritional supplements such as vitamins or minerals, or were pregnant or lactating. MS patients who were using drugs to treat clinical features of cardiovascular diseases such as blood pressure, were using lipid- and/or glucose-lowering drugs, or did not relay the full details of their food consumption were also excluded from the study. The study was approved by the Ondokuz Mayıs University Clinical Research Ethics Committee (decision number B.30.2.ODM.0.20.08/255) and the principles of the Declaration of Helsinki were applied.

### 2.2. Research instruments

A questionnaire form addressing demographic characteristics was administered at the first meeting with all patients. Characteristics including age, sex, MS duration, marital status, education level, and smoking status of the patients were determined with this questionnaire form. In addition, the Expanded Disability Status Scale (EDSS) scores of the patients were evaluated by a neurologist during patient examinations.

### 2.3. Anthropometric measurements and body composition

All anthropometric measurements were taken by a research dietician using calibrated instruments. Height was measured with a portable height meter, with the head in an upright position and eyes facing forward, with the patient standing and fixed in the Frankfort plane. Body weight was measured with light clothes and without shoes on an empty stomach in the morning [33]. Body composition [body fat percentage, BFM, and body fat-free mass (BFFM)] was measured using bioelectrical impedance analysis (BC-418 model, Tanita, Tokyo, Japan). Waist circumference (WC, in cm) and hip circumference (HC, in cm) were measured using a nonelastic tape with precision of 0.1 cm. BMI was calculated using the formula of weight (kg)/height (m)^2^ and BMI classifications were performed based on the guidelines of the World Health Organization for adults [34]. According to these guidelines, participants were categorized as underweight (<18.5 kg/m^2^), normal weight (≥18.5 to <25 kg/m^2^), overweight (≥25 to <30 kg/m^2^), or obese (≥30 kg/m^2^). Waist-hip ratio (WHR) was calculated using the formula of waist circumference (cm)/hip circumference (cm) and waist-height ratio was calculated as waist circumference (cm)/height (cm).

### 2.4. Biochemical parameters

The results of the routine blood tests requested by the physician during the examination were evaluated. From each participant, a 10-mL blood sample was collected in tubes containing ethylenediaminetetraacetic acid (EDTA). After collection, blood plasma and buffy coat were separated by centrifugation. Total cholesterol (TC), triglyceride (TG), and high-density lipoprotein cholesterol (HDL-c) were analyzed enzymatically with autoanalyzers and the low-density lipoprotein cholesterol (LDL-c) formula of Friedewald was applied. Results were reviewed via the institution’s patient system.

### 2.5. Dietary intake assessment

In order to determine individuals’ food and nutrient intake, a prospective 3-day 24-h food consumption record (1 day on the weekend and 2 weekdays) was used [35]. Nutritionists asked the patients about their food consumption. Patients were asked to write all the foods and amounts they consumed for 24 h. Before the form was administered, brief information was given about the quantities and contents of foods using a food and food photo catalogue [36]. The computer-aided Nutrition Information Systems Package Program (BeBiS) was used to calculate the contents of the nutrients reflected by the records of all patients.

### 2.6. Calculation of DII scores

DII scores were calculated to determine the inflammatory contents of the patients’ diets. The calculation of the DII in this study sample was based on the methodology proposed and validated by Shivappa et al. [27]. The DII was created based on a systematic review of studies investigating the effects of various food ingredients (i.e., 45 food parameters) on specific proinflammatory and inflammatory biomarkers [i.e., interleukin (IL)-10, IL-4, IL-6, IL-1β, tumor necrosis factor (TNF)-α, and C-reactive protein (CRP)]. A particular z-score for each food, as described previously, was calculated, and the z-score of each food component was converted to the averaged percentage score for each patient and then multiplied by the specific food material effect score. With this procedure, the specific food component DII score of each patient was calculated. Finally, the DII scores of all specific food ingredients were summed and the total DII score for each patient was generated. In the evaluation of this index, higher DII scores indicate proinflammatory diets and lower scores indicate antiinflammatory diets [27].

In this study, 36 of the possible 45 parameters were used to calculate the DII score, including a number of nutrients, specific food items, and bioactive compounds. These were energy, carbohydrates, total fat, protein, fiber, cholesterol, monounsaturated fatty acid (MUFA), polyunsaturated fatty acid (PUFA), saturated fatty acid (SFA), folic acid, niacin, riboflavin, thiamin, vitamin A, vitamin C, vitamin D, vitamin E, β-carotene, zinc, selenium, magnesium, iron, caffeine, pepper, onion, garlic, green/black tea, vitamin B12, vitamin B6, alcohol, ginger, omega-3 fatty acids, omega-6 fatty acids, trans fat, and saffron.

### 2.7. Statistical analysis

Histograms, q-q plots, and the Shapiro–Wilk test were examined to assess the normality of the data. The Levene test was used to evaluate variance homogeneity. DII groups were created using tertiles. To compare differences among the patients’ characteristics, anthropometric measurements, and lipid profiles among the DII groups, either one-way analysis of variance (ANOVA) or the Kruskal–Wallis H test was applied for continuous variables, while Pearson chi-square analysis was applied for categorical variables. Tukey, Tamhane T2, and Bonferroni adjusted z-tests were performed for multiple comparisons. To examine the relationships between variables, Spearman rho correlation coefficients were also calculated. These coefficients were interpreted as follows: 0–0.30, very weak correlation; 0.31–0.50, weak correlation; 0.51–0.70, moderate correlation; 0.71–0.90, high correlation; and 0.91–1.00, very high correlation. Moreover, linear regression analysis was conducted to identify the crude and adjusted effects of anthropometric measurements and lipid profiles on DII scores. Adjusted models were built by controlling the effects of age and sex in Model I and smoking status, education level, marital status, MS duration, EDSS score, and energy intake in Model II. Standardized regression coefficients (beta), standard errors, and p-values were summarized and p-values of less than 5% were considered statistically significant. All analyses were conducted using R 3.5.1 (www.r-project.org) and TURCOSA (www.turcosa.com.tr) software.

## 3. Results

In this study, a total of 85 patients with MS were included. The average age of the patients was 35.80 ± 10.49 years. Detailed information about patient characteristics among the tertiles of the DII scores is presented in Table 1. The patients were divided into T_1_ (−4.26 to −0.29), T_2_ (−0.28 to 0.98), and T_3_ (1.01 to 3.58) tertiles according to their DII scores. As seen in Table 1, the age, MS duration, EDSS score, BMI classification, smoking status, alcohol use, marital status, and education level of the patients did not differ among the tertiles (p > 0.05). However, the energy intake of the patients differed among the tertiles (p < 0.05). Patients in the group with higher DII scores, reflecting a more proinflammatory diet, tended to have higher energy intake. The education levels of the patients did not differ between the groups, but a significant difference was found for the DII scores of those who received undergraduate education (p < 0.05).

Table 2 shows the dietary intake data of patients and comparisons with the global dietary intake data of Shivappa et al. [27]. Compared to the global averages, patients in this study had relatively high intake of proinflammatory contents such as protein and total fat. They had relatively low intake of antiinflammatory contents such as alcohol, β-carotene, caffeine, folic acid, garlic, ginger, magnesium, MUFA, niacin, onion, saffron, selenium, thiamin, vitamin C, vitamin D, and zinc but relatively high intake of other antiinflammatory parameters such as fiber, vitamin B6, omega-3 fatty acids, omega-6 fatty acids, PUFA, vitamin A, vitamin E, green/black tea, and pepper.

Table 3 shows the comparisons of anthropometric measurements and lipid profiles among the tertiles of DII scores. The mean percentages of body fat differed among the DII tertiles (p < 0.05). Body fat percentages were higher in the group with high DII scores. Body fat percentage had a positive and very weak correlation with DII scores (rho = 0.206 and rho = 0.217, respectively). The mean percentages of HDL-c also differed among the DII tertiles (p < 0.05). HDL-c was higher in the group with high DII scores. A positive and weak correlation was observed between HDL-c and DII scores (rho = 0.307, p < 0.05).

Table 4 shows the crude and adjusted linear regression models used to identify the relationships between DII scores, anthropometric measurements, and lipid profiles. As seen in Table 4, only the effect of HDL-c on the DII was significant in the crude model, both before and after controlling for potential confounders (Model I: age and sex; Model II: smoking status, education level, marital status, MS duration, EDSS, and energy intake) (p < 0.05).

## 4. Discussion

The DII is a relatively novel emerging global tool to evaluate the inflammatory potential of the diet, reflecting the standardization of individual dietary references to global reference values [27]. Results based on this index show that it reliably predicts concentrations of inflammatory markers such as IL-1β, IL-4, IL-6, IL-10, TNF-α, and C-reactive protein [27,30]. In this study, high DII scores were associated with increased weight, HC, body fat percentage, BFM, BMI, and lipid profiles and decreased BFFM. A proinflammatory diet, as indicated by a higher DII score, is associated with increased odds of elevated body fat percentage. The DII score can thus be associated with the inflammatory potential of the diet and obesity in MS patients, and it can be used from a clinical point of view for assessment.

In this study, it was observed that DII scores increased as the level of education increased (Table 1). The reason for this may be that people with higher levels of education are exposed to proinflammatory diets due to their employment status. This may also be more specifically related to the role of physical activity, as patients with less education may have jobs that are more physically demanding in comparison to more educated patients, and healthy antiinflammatory diets together with higher levels of work-related physical activity could be associated with reductions of obesity and BMI [37].

In this study, we also compared the intake of DII components by MS patients with the global averages described by Shivappa et al. [27] (Table 2). The DII scores of the patients were between −4.26 and 3.58. Compared to global averages for MS patients, these patients consumed more total fat and protein, which are proinflammatory contents, while they consumed less alcohol, β-carotene, caffeine, folic acid, garlic, ginger, magnesium, MUFA, niacin, onion, saffron, selenium, thiamin, vitamin C, vitamin D, and zinc, which are antiinflammatory dietary contents. In the study conducted by Muhammad et al. in an Indonesian population, it was found that participants had higher levels of intake of proinflammatory components such as total energy, carbohydrates, iron, and vitamin B12 and lower intake of antiinflammatory components such as omega-3 fatty acids, omega-6 fatty acids, niacin, vitamin A, vitamin D, and vitamin E [38].

In the cross-sectional CUME project (Brazilian Graduates from the Cohort of Universities of Minas Gerais), which evaluated 3151 adolescents, Oliveira et al. reported that individuals in the most proinflammatory DII quartile had higher levels of consumption of red, fatty, and ultraprocessed meats as well as fats, soft drinks, sugar, and sweets, while those in the most antiinflammatory DII quartile had higher consumption of dairy products, white and lean meats, fish/shellfish, eggs, whole grains, legumes, olive oil, fruits, and vegetables [39]. Another cross-sectional study, the HELENA (Healthy Lifestyle in Europe) study, conducted with 3528 adolescents, found that increased DII scores were associated with higher consumption of bread, chocolate, margarine, butter, animal fats, vegetable lipids, acidic items, soft drinks, meat, cakes, pies, biscuits, and sugar/honey/jam, while decreased DII scores were associated with higher consumption of vegetables (excluding potatoes), fruit, fruit and vegetable juices, and fish [40]. Dietary patterns are among the important variables that can be used to try to explain relationships between diets and diseases, constituting an effective tool in evaluating the inflammatory potential of the diet while taking into account the potential synergies of different foods [41]. Given this, it is obvious that individuals with antiinflammatory diets tend to have healthier weights. In calculating the DII score, a total of 45 parameters are used, 8 of which are proinflammatory parameters and 37 of which are antiinflammatory parameters. The latter group of antiinflammatory parameters comprises food components characterized by being colorful, tasty, and low-calorie [27]. Therefore, consuming these components will not only reduce inflammation but is also likely to lead to weight loss.

In this study, it was observed that as DII scores increased, weight, WC, HC, body fat percentage, BFM, and BMI values increased. In addition, in the last tertile, body fat percentage was significantly increased (Table 3). While body fat percentage was found to be significantly different between the inflammatory index tertile groups (p < 0.05), body fat mass did not differ significantly (p > 0.05). When the Spearman correlation coefficients were examined, it was observed that both of these variables were very weakly correlated with the DII. The discrepancy between these two variables is due to the fact that the coefficient of variation for BFM is higher compared to the coefficient of variation for body fat percentage. The statistical power was not sufficient due to the higher variation in BFM and no difference was observed between the mean values of the inflammatory index tertile groups. However, the correlation coefficient, which is a measure of effect size, was not affected by the sample size and demonstrated a very weak relationship between the variables. Similarly, the PREDIMED study, which included 7236 Spanish people, showed that diets with more proinflammatory contents (i.e., higher DII scores) had positive relationships with BMI, WC, and WHR. These findings confirmed the relationship between obesity and inflammation [42], which releases many adipokines and inflammatory cytokines that play a role in adipose tissue, immunity, and inflammatory processes [43]. On the other hand, according to recent hypotheses, mild and chronic inflammation can lead to obesity [42].

In this study, as DII scores increased, TG and LDL-c increased, but DII scores were not correlated with components of the lipid profile such as TC, TG, and LDL-c (Table 3). DII scores were also positively associated with HDL-C levels before and after controlling for potential confounders (Tables 3 and 4). There is an important relationship between inflammation and cardiovascular comorbidities [44]. A small-scale study of 90 adults performed by Camargo-Ramos et al. found that lower DII scores (i.e., antiinflammatory diets) were significantly associated with higher HDL-c and inversely correlated with plasma TG levels [45]. A French cohort study of 3726 adults performed by Neufcourt et al. initially showed that DII scores were positively related to TG levels but not to HDL-c levels [46]. The reason for observing a positive relationship between DII scores and HDL-c could be that patients in the proinflammatory group had begun calorie-restricted diets, consuming healthier foods and increasing their physical activity. As seen in Table 1, the energy intake of the patients in tertile T_3_ was less than that of the other tertiles. Similarly, a clinical trial with 45 volunteers conducted by Ferreira et al. found that DII scores were positively associated with HDL-c after a weight loss program [47]. Another explanation is that inflammation may lead to dysfunctional HDL-c formation due to oxidative damage. HDL particles are susceptible to structural modifications mediated by various mechanisms, including oxidation, glycation, and enzymatic degradation, affecting their functional properties. Moreover, in vitro studies have shown that the homocysteinylation of HDL may reduce the activity of the PON enzyme, which is associated with human HDL-c, thus rendering HDL particles more susceptible to oxidative damage. Formation of inflammatory HDL has been suggested to correlate with decreases in the activities of various HDL-associated enzymes, such as PON, a multifunctional enzyme with antioxidant capacity and the ability to detoxify the homocysteine metabolite homocysteine thiolactone [48–50]. It is also known that the immunomodulatory drugs used by MS patients affect their biochemical results [51,52].

In conclusion, we have shown that DII scores may be associated with the inflammatory potential of the diet and obesity in MS patients and can be used from a clinical point of view for assessment. Due to the potential importance of the diet in the development of inflammation, studies with larger samples and intervention studies are needed to investigate the effects of the manipulation of the inflammatory properties of the diet on inflammatory processes in patients with MS. In studies with larger samples, the relationships between the DII and immunological profiling, MRI findings, and the EDSS should be evaluated in MS patients.

This study has yielded several important results. To the best of our knowledge, this study is the first to examine the relationships between the DII and BMI, fat percentage, BFM, and lipid profiles in MS patients. However, some limitations must be noted. First, our study was cross-sectional; therefore, no conclusions about causality can be drawn [53]. Second, data collection was done with limited data on the bioactive components of foods and we cannot provide information on the intake of a number of herbs and spices (e.g., oregano, rosemary, and turmeric) or polyphenols (e.g., flavonol, anthocyanidins, and eugenol). Third, we did not ask about calorie restrictions or physical activity status while choosing patients for the study. Finally, the DII scores of MS patients and healthy individuals could be compared in future studies by taking healthy individuals as the control group.

## Figures and Tables

**Table 1:** Comparisons of patient characteristics among DII score tertiles.

Patient characteristics	T_1_ (n = 28)	T_2_ (n = 28)	T_3_ (n = 29)	Total	p
Age (years)	36.43 ± 10.16	36.46 ± 11.14	34.55 ± 10.43	35.80 ± 10.49	0.737
MS duration (years)	4.5 (2.0–9.0)	4.0 (2.0–12.0)	5.0 (2.0–12.0)	4.0 (2.0–11.0)	0.858
EDSS score	0.0 (0.0–1.9)	0.5 (0.0–1.9)	0.0 (0.0–1.3)	0.0 (0.0–1.5)	0.736
Energy intake	2021.01 ± 471.12^a^	1776.38 ± 424.86^a^	1446.22 ± 448.09^b^	1744.32 ± 502.82	<0.001
BMI classification					
Underweight (<18.50 kg/m^2^)	0 (0.0)	2 (7.1)	0 (0.0)	2 (2.4)	0.285
Normal (20.0–25.0 kg/m^2^)	14 (50.0)	12 (42.9)	10 (34.5)	36 (42.4)	
Overweight (25.1–29.9 kg/m^2^)	7 (25.0)	7 (25.0)	13 (44.8)	27 (31.8)	
Obese (≥30.0 kg/m^2^)	7 (25.0)	7 (25.0)	6 (20.7)	20 (23.5)	
Smoking status (yes)	5 (17.9)	9 (32.1)	6 (20.7)	20 (23.5)	0.410
Alcohol use (yes)	0 (0.0)	1 (3.6)	0 (0.0)	1 (1.2)	0.357
Marital status (married)	21 (75.0)	21 (75.0)	22 (75.9)	64 (75.3)	0.996
Education level					
Primary school	14 (50.0)^a^	10 (35.7)^a^	7 (24.1)^a^	31 (36.5)	0.044
High school	10 (35.7)^a^	12 (42.9)^a^	11 (37.9)^a^	33 (38.8)	
Undergraduate	2 (7.1)^a^	6 (21.4)^a^^b^	11 (37.9)^b^	19 (22.4)	
Graduate	2 (7.1)^a^	0 (0.0)^a^	0 (0.0)^a^	2 (2.4)	

Values are expressed as n (%), mean ± SD, or median (first-third quartiles). T_i_ denotes the i^th^ tertile. Different bold superscripts in the same row indicate statistically significant differences among groups. BMI: Body mass index; EDSS: Expanded Disability Status Scale.

**Table 2:** Comparison of global dietary intake and dietary intake of the current study’s MS patients.

Intake	Dietary intake of study participants	Global dietary intake
Proinflammatory parameters		
Carbohydrate (g)	192.76 ± 67.87	272.2 ± 40.0
Cholesterol (mg)	249.09 ± 111.98	279.4 ± 51.2
Energy (kcal)	1744.32 ± 502.82	2056 ± 338
Iron (mg)	10.73 ± 3.58	13.35 ± 3.71
Protein (g)	83.61 ± 36.51	79.4 ± 13.9
Saturated fat (g)	25.17 ± 8.94	28.6 ± 8.0
Total fat (g)	79.65 ± 23.01	71.4 ± 19.4
Trans fatty acid (g)	0 ± 0	3.15 ± 3.75
Vitamin B12 (μg)	3.48 ± 3.07	5.15 ± 2.70
Antiinflammatory parameters		
Alcohol (g)	0.05 ± 0.18	13.98 ± 3.72
β-carotene (μg)	2610 ± 1959.92	3718 ± 1720
Caffeine (g)	1.08 × 10^−3^ ± 2.41 × 10^−3^	8.05 ± 6.67
Fiber (g)	21.02 ± 7.09	18.8 ± 4.9
Folic acid (μg)	208.08 ± 118.52	273.0 ± 70.7
Garlic (g)	0.2 ± 0.56	4.35 ± 2.90
Vitamin B6 (mg)	1.81 ± 1.21	1.47 ± 0.74
Ginger (g)	0 ± 0	59.0 ± 63.2
Magnesium (mg)	240.77 ± 81.31	310.1 ± 139.4
MUFA (g)	25.27 ± 7.97	27.0 ± 6.1
Niacin (mg)	23.02 ± 14.31	25.9 ± 11.77
Omega-3 fatty acids (g)	2.35 ± 1.67	1.06 ± 1.06
Omega-6 fatty acids (g)	21.42 ± 7.86	10.80 ± 7.50
Onion (g)	31.43 ± 75.51	35.9 ± 18.4
PUFA (g)	23.88 ± 8.38	13.88 ± 3.76
Saffron (g)	0 ± 0	0.37 ± 1.78
Selenium (μg)	0.15 ± 0.42	67.0 ± 25.1
Thiamin (mg)	0.85 ± 0.27	1.70 ± 0.66
Vitamin A (RE)	1237.67 ± 983.79	983.9 ± 518.6
Vitamin C (mg)	102.73 ± 64.85	118.2 ± 43.46
Vitamin D (μg)	4.51 ± 10.98	6.26 ± 2.21
Vitamin E (mg)	24.16 ± 8.28	8.73 ± 1.49
Zinc (mg)	5.78 ± 3.98	9.84 ± 2.19
Green/black tea (g)	6.6 ± 3.35	1.69 ± 1.53
Pepper (g)	13.14 ± 19.46	10.0 ± 7.07

MUFA: Monounsaturated fatty acid; PUFA: polyunsaturated fatty acid; RE: retinol equivalent.

**Table 3:** Comparison of anthropometric measurements and lipid profiles among DII score tertiles.

Anthropometric measurements	T_1_ (n = 28)	T_2_ (n = 28)	T_3_ (n = 29)	Total	Spearman ρ	p
Height (m)	1.66 ± 0.09	1.65 ± 0.09	1.63 ± 0.07	1.65 ± 0.08	−0.071	0.599
Weight (kg)	71.18 ± 16.90	71.96 ± 17.83	73.71 ± 16.85	72.30 ± 17.02	0.084	0.850
WC (cm)	88.52 ± 13.65	89.34 ± 21.00	89.36 ± 14.98	89.08 ± 16.63	0.034	0.977
HC (cm)	103.66 ± 10.74	105.25 ± 11.91	107.29 ± 10.68	105.42 ± 11.09	0.166	0.468
WHR	0.85 ± 0.07	0.84 ± 0.10	0.83 ± 0.09	0.84 ± 0.09	−0.116	0.672
Waist-height ratio	0.54 ± 0.08	0.54 ± 0.12	0.55 ± 0.09	0.54 ± 0.10	0.057	0.915
Body fat (%)	26.44 ± 11.57^a^	27.60 ± 12.82^a^^b^	32.67 ± 7.79^b^	28.95 ± 11.12	0.206*	**0.039** *
BFM (kg)	19.58 ± 11.35	21.21 ± 14.47	25.00 ± 11.17	21.97 ± 12.47	0.217*	0.243
BFFM (kg)	51.57 ± 11.31	50.74 ± 9.35	48.86 ± 8.75	50.37 ± 9.80	−0.062	0.568
BMI (kg/m^2^)	25.99 ± 5.61	26.36 ± 6.11	27.51 ± 5.70	26.63 ± 5.77	0.120	0.590
Lipid profiles						
HDL-c (mg/dL)	48.47 ± 10.52^a^	52.74 ± 10.69^a^^b^	58.63 ± 13.56^b^	53.34 ± 12.30	0.307*	**0.006** *
LDL-c (mg/dL)	93.69 ± 33.60	94.94 ± 40.06	99.17 ± 40.71	95.97 ± 37.90	0.062	0.852
TC (mg/dL)	173.54 ± 42.02	175.23 ± 48.99	178.69 ± 45.79	175.85 ± 45.20	0.076	0.910
TG (mg/dL)	121.1 (77.9–215.6)	114.7 (87.3–168.0)	101.3 (72.5–150.0)	110.0 (81.1–176.3)	−0.087	0.655

* p < 0.05.

Values are expressed as mean ± SD. T_i_ denotes the i^th^ tertile. Different bold superscripts in the same row indicate statistically significant differences among groups. BFM: Body fat mass; BFFM: body fat-free mass; BMI: body mass index; HC: hip circumference; WC: waist circumference; WHR: waist-hip ratio; HDL-c: high-density lipoprotein cholesterol; LDL-c: low-density lipoprotein cholesterol, TG: triglyceride, TC: total cholesterol.

**Table 4:** Crude and adjusted linear regression models for identifying the relationships between the DII, anthropometric measurements, and lipid profiles.

Variables	Model 0^a^			Model I^b^			Model II^c^		
	Beta	SE	p	Beta	SE	p	Beta	SE	p
Anthropometric measurements									
Height (m)	−0.075	1.893	0.496	0.197	2.364	0.153	0.157	1.892	0.154
Weight (kg)	0.057	0.009	0.607	0.116	0.009	0.281	0.121	0.009	0.228
WC (cm)	0.014	0.010	0.901	0.065	0.010	0.559	0.086	0.009	0.404
HC (cm)	0.139	0.014	0.206	0.129	0.014	0.232	0.160	0.013	0.121
WHR	−0.107	1.813	0.328	−0.003	1.938	0.978	−0.025	1.691	0.807
Waist-height ratio	0.036	1.638	0.744	0.036	1.678	0.746	0.045	1.575	0.669
Body fat (%)	0.205	0.014	0.060	0.068	0.019	0.632	0.085	0.015	0.466
BFM (kg)	0.171	0.013	0.119	0.097	0.014	0.408	0.130	0.012	0.224
BFFM (kg)	−0.111	0.016	0.310	0.185	0.021	0.201	0.072	0.016	0.490
BMI (kg/m^2^)	0.101	0.028	0.357	0.082	0.028	0.455	0.069	0.027	0.516
Lipid profiles									
HDL-c (mg/dL)	0.306	0.012	**0.004** *	0.251	0.012	**0.019** *	0.215	0.011	**0.025** *
LDL-c (mg/dL)	0.109	0.004	0.322	0.193	0.005	0.121	−0.054	0.004	0.630
TC (mg/dL)	0.074	0.004	0.499	0.142	0.004	0.240	−0.073	0.003	0.498
TG (mg/dL)	−0.151	0.002	0.168	−0.107	0.002	0.345	−0.119	0.002	0.246

a Model 0: Linear regression analysis without adjustment;

b Model I: linear regression analysis with adjustment for age and sex;

c Model II: linear regression analysis with correction for smoking status, education level, marital status, MS follow-up time, EDSS, and energy intake.

* p < 0.05.

BFM: Body fat mass; BFFM: body fat-free mass; BMI: body mass index; HC: hip circumference; WC: waist circumference; WHR: waist-hip ratio; HDL-c: high-density lipoprotein cholesterol; LDL-c: low-density lipoprotein cholesterol, TG: triglyceride, TC: total cholesterol, EDSS: Expanded Disability Status Scale.
